# Mapping Covariation Quantitative Trait Loci That Control Organ Growth and Whole-Plant Biomass

**DOI:** 10.3389/fpls.2019.00719

**Published:** 2019-06-04

**Authors:** Jingwen Gan, Yige Cao, Libo Jiang, Rongling Wu

**Affiliations:** ^1^Center for Computational Biology, College of Biological Sciences and Technology, Beijing Forestry University, Beijing, China; ^2^Center for Statistical Genetics, Departments of Public Health Sciences and Statistics, The Pennsylvania State University, Hershey, PA, United States

**Keywords:** functional mapping, growth equation, covariation, *Arabidopsis thaliana*, quantitative trait locus (QTL)

## Abstract

Covariation between organ growth and biomass accumulation plays an important role in plants. Plant to capture optimal fitness in nature, which depend coordinate and interact for distinct organs such as leaves, stems, and roots. Although many studies have focused on plant growth or biomass allocation, detailed information on the genetic mechanism of coordinated variation is lacking. Here, we expand a new mapping model based on functional mapping to detect covariation quantitative trait loci (QTLs) that govern development of plant organs and whole biomass, which, via a series of hypothesis tests, allows quantification of how QTLs regulate covariation between organ growth and biomass accumulation. The model was implemented to analyze leaf number data and the whole dry weight of recombinant inbred lines (RILs) of *Arabidopsis*. Two key QTLs related to growth and biomass allocation that reside within biologically meaningful genes, *CRA1* and *HIPP25*, are characterized. These two genes may control covariation between two traits. The new model will enable the elucidation of the genetic architecture underlying growth and biomass accumulation, which may enhance our understanding of fitness development in plants.

## Introduction

A plant includes multiple organs, each of which serves different functions; for example, leaves photosynthesize organic compounds, and roots take up water and nutrients. Plant to capture optimal fitness in nature, which depend coordinate and interact for distinct organs (Carbone et al., 2013). The whole biomass of a plant is the cumulative result of dynamic carbon allocation and the rate of mass loss for organ growth and development during its life cycle (Poorter et al., 2015). Several gene-mapping studies have investigated the genetic mechanisms by which plant biomass and organ growth are controlled (Lisec et al., 2008; Atkinson et al., 2015; Jiang et al., 2015; Song et al., 2015). In addition, research on biomass allocation has revealed an ecologic relationship between structure and function (Chen and Reynolds, 1997; Weiner, 2004). For example, the relationship between leaf area and plant biomass is non-linear and dynamic and depends on carbon accumulation and partition in *Arabidopsis thaliana* (Weraduwage et al., 2015). However, little is known of the genetic mechanisms underlying covariation between organ growth and whole-plant biomass.

Genetic mapping enables characterization of QTL that effect complex traits. The genetic-mapping approaches developed to date include interval mapping, composite interval mapping, and multiple interval mapping (Lander and Botstein, 1989; Zeng, 1994; Kao et al., 1999). The development of high-throughput sequencing technology has enabled genome-wide association studies (GWASs) of the associations between single-nucleotide polymorphisms (SNPs) and phenotype (Kruglyak, 2008; Civelek and Lusis, 2014). However, these methods are static and do not take into consideration the developmental pattern of phenotype. Ma et al. proposed a dynamic model for mapping growth QTLs for a complex trait. This model, known as functional mapping, which incorporates growth equations into genetic mapping is capable of addressing fundamental issues in quantitative genetics and developmental biology (Ma et al., 2002; Wu and Lin, 2006). However, for QTL mapping, GWAS or functional mapping cannot map the covariation between organ growth and whole-plant biomass, which cannot simultaneously consider dynamic and static phenotype, respectively.

In this study, we attempt to extend a mapping model-functional mapping-derived which incorporate the static feature into the dynamic one to detect covariation QTLs that govern the development of plant organs and the increase in whole-plant biomass. Our model capitalizes on QTL genotype-dependent growth parameters estimated from functional mapping and enables hypothesis testing for the genetic effects of covariation QTLs. Here, we show how the model can be used to map QTLs associated with leaf growth and whole biomass of the model plant *A. thaliana*. To evaluate the performance of our model, we perform a computer simulation based on the results of data analyses and compare functional mapping with the traditional method for dynamic and static data, respectively. The new model enables mapping of covariation genes and will enhance our understanding of the effects of these genes on plant growth and biomass accumulation.

## Model

### Statistical Model

Consider a mapping or natural population that it is composed of *n* individuals, each genotyped for a panel of molecular markers. A growth trait of each individual *i*, such as plant height or leaf number, is measured at *T* time points. After the end of the life cycle, the whole-plant biomass of each individual plant was determined. Because high-throughput sequencing markers cover the entire genome, we employed the multiplicative model for mapping and for natural populations.

Let *y*_*i*_ = (*y*_*i*_(1), …, *y*(T)) and *z*_*i*_ denote the vectors of time-dependent growth trait values and biomass, respectively, of individual *i*. We employed the multiplicative model, which assumes that QTLs are located at the marker positions. We hypothesized that growth traits and biomass accumulation are controlled by a set of QTLs throughout the genome, such that the multiplicative likelihood model for each marker is expressed as:

(1) $$\begin{array}{l} L\left(\Omega |Y\right)=\overset{J}{\underset{j=1}{\prod }}\overset{n_{j}}{\underset{i=1}{\prod }}f_{j}\left(Y_{i}|\mu_{j},\Sigma \right) \end{array}$$

where Ω = *c*(μ_*j*_, Σ) is the unknown parameter set; *Y*_*i*_ = *c*(*y*_*i*_, *z*_*i*_) is the vector of individual *i* including the growth trait y and biomass index z; *J* is the number of genotypes for each marker; *n*_*j*_ is the number of individuals with marker genotype *j* and *f*_*j*_(*Y*_*i*_|μ_*j*_, Σ) is the multivariate normal density function with the genotype-dependent mean vector:

(2) $$\begin{array}{l} u_{j}=c\left(\mu_{j}^{y},\mu_{j}^{z}\right)=c\left(\mu_{j}^{y}\left(1\right),\ldots ,\mu_{j}^{y}\left(T\right),\mu_{j}^{z}\right) \end{array}$$

and the covariance matrix:

(3) $$\begin{array}{l} \Sigma =\left(\begin{array}{cc} \Sigma_{y} & \Sigma_{yz}\\ \Sigma_{zy} & \sigma_{z}^{2} \end{array}\right) \end{array}$$

where $\mu_{j}^{y}$ are the expected means of the growth trait for genotype *j* from time 1 to *T*; $\mu_{j}^{z}$ are the expected means of the static trait for genotype *j*; Σ_*y*_ and $\sigma_{z}^{2}$ are the covariance matrix and variance for the growth and static traits, respectively, and $\Sigma_{yz}=\Sigma_{zy}^{{\;}^{\prime }}$ being a (*T* × 1) covariance matrix between the two traits.

### Modeling the Mean Vector

Because we analyzed dynamic and static data, we reconstructed the mean vector. For a growth trait, the time-dependent genotypic values for different QTLs can be fitted to a logistical growth equation, which is expressed as:

(4) $$\begin{array}{l} g\left(t\right)=\frac{a}{1+be^{-rt}} \end{array}$$

where *g*(*t*) is the trait value at time *t*; *a* is the asymptotic value of g when *t* tends to be infinite; *b* describes the initial growth; and *r* is the relative growth rate. Functional mapping does not estimate the expected genotypic means using (Equation 2), but models these means using (Equation 4). Specifically, the mean value vector (2) for genotype *j* is expressed as:

(5) $$\begin{array}{l} \mu_{j}=c\left(\mu_{j}^{y},\mu_{j}^{z}\right)=c\left(\frac{a_{j}}{1+b_{j}e^{-r_{j}}},\ldots ,\frac{a_{j}}{1+b_{j}e^{-r_{j}T}},\mu_{j}^{z}\right) \end{array}$$

through a set of parameters $\left(a_{j},b_{j},r_{j},\mu_{j}^{z}\right)$. Thus, we can test whether this QTL covariate affects growth and biomass allocation by comparing differences in the genotype-dependent parameter sets.

### Modeling the Covariance Matrix

In Equation (3), Σ_*y*_ is the covariance matrix for longitudinal traits. We used a cost-effective method to model the longitudinal covariance matrix by a particular parameter set. To date, several methods, such as autoregressive, antedependence, and non-parametric methods, have been used to describe this matrix (Ma et al., 2002; Zhao et al., 2005; Yap et al., 2009). Of these, auto regression may be the most parsimonious as it uses fewer parameters to capture the complex structure of a matrix and exemplifies the statistical power of QTL mapping. The construction of Σ_*y*_ can be modeled by the most flexible first-order autoregressive (*AR*(1)) model in which there are two parameters; i.e., there is the same residual variance (σ_*y*_) for the trait at each time point, and the stationary covariance, which decreases proportionally (in ρ_*y*_) with increased interval between measurements. The covariance matrix Σ_*yz*_ is composed of the correlation coefficient ϕ and variance $\sigma_{z}^{2}$ of the static trait. The parameter φ is used to measure the correlation between dynamic and static data. Thus, the covariance matrix Σ can be explicitly expressed by $\left(\sigma_{y}^{2},\rho_{y},\varphi ,\sigma_{z}^{2}\right)$. In this study, we employed a simplex algorithm to estimate all parameters by optimizing (Equation 1). The genotype and covariance parameters are estimated separately for each marker.

### Hypothesis Tests

The existence of a significant QTL can be tested by the following hypothesis tests:

(6) $$\begin{array}{l} H_{0}:\left(a_{j},b_{j},r_{j},\mu_{j}^{z}\right)\equiv \left(a,b,r,\mu^{z}\right)\;forj=1,\ldots ,J\\ H_{1}:Not\;all\;equalities\;above\;do\;not\;hold \end{array}$$

where *H*_0_ corresponds to the reduced model in which no QTL affects growth and biomass accumulation, and *H*_1_ corresponds to the full model in which QTLs exist. We calculated the log-likelihood ratio (LR) of the reduced to the full model and comparing it with a critical threshold. The threshold can be empirically determined from permutation tests by reshuffling the phenotypic data 1,000 times (Churchill and Doerge, 1994). The maximum LR value was extracted for each sampling. The top 5% of LR values were used as a critical threshold to indicate a significance level of 0.05.

After a QTL was significant, we tested its effect on organ growth. We formulated the following null hypotheses:

(7) $$\begin{array}{l} H_{0}:\left(a_{j},b_{j},r_{j}\right)\equiv \left(a,b,r\right)\;for\;j=1,\ldots ,J \end{array}$$

We also tested whether the QTLs affect biomass accumulation by formulating the following null hypotheses:

(8) $$\begin{array}{l} H_{0}:\mu_{j}^{z}\equiv \mu^{z}\;for\;j=1,\ldots ,J \end{array}$$

The above three tests are used to determine whether a QTL is a covariation QTL.

## Application

### Mapping Population

We obtained a mapping population composed of 116 recombinant inbred lines (RILs) derived from *A. thaliana* Landsbergerecta (Ler) and Shakdara (Sha). These RILs were genotyped by resequencing technology, producing 417,495 good-quality polymorphic SNPs (Jiang et al., 2018). Each RIL was grown in a square plate filled with a 1:1:1 mixture of vermiculite + turfy soil + limestone. All seeds germinated normally into 5,220 seedlings at 25°C, humidity 70%, and 8 h of light at 25 Wm^−2^ in a climate chamber. Starting 1 week after seed sowing, the number of leaves of a set of 10 plants from each RIL was measured weekly until lifecycle completion. Whole plants (including roots) were collected after the last phenotypic measurement and transferred to an electrothermal constant-temperature dry box at 105°C for 48 h. Finally, the dry weight of each individual plant was measured.

### QTL Mapping

To evaluate the applicability of growth equations to characterizing the development pattern of a trait, a least-squares approach was used to fit leaf number over time with the logistical curve of each individual. The dynamic change in leaf number was well fitted by the logistical curve (Equation 4) for each RIL (*R*^2^ > 0.96; Figure S1). In addition, the different growth trends of the RILs implies genetic control of the growth in the number of leaves. The statistical model based on the logistical growth curve was used to map the QTLs responsible for changes in leaf number.

**Figure 1:**
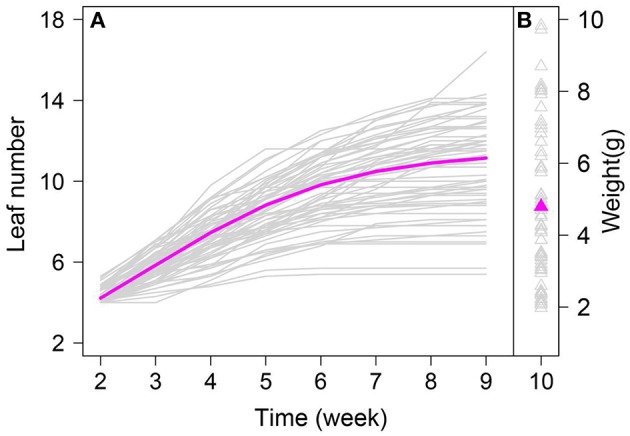
Leaf number change trajectories in an RIL of *Arabidopsis*, fitted by a logistic equation. Magenta line represents the overall growth curve fitted to leaf number of all RILs (Gray line). Magenta triangle represents the mean fitted to dry weight of all RILs (Gray triangle).

We fitted the mean growth curve of leaf number and mean dry weight for all *A. thaliana* individuals based on the new model. Figure 1A shows the mean growth curve fitted by Equation (4), from which we identified the growth in leaf number over time. The rate of increase in leaf number at the early stage was faster than that subsequently, and leaf number exhibited asymptotic growth during whole growth period (Figure 1A). The fitted mean value for dry weight, a static trait measured until lifecycle completion, was 5.05, which reflects biomass accumulation in *A*. *thaliana* (Figure 1B). By estimating φ, we quantified the correlation between dry weight and leaf number. There was a significant correlation between the growth in leaf number and in biomass in *A*. *thaliana* (*P* < 0.01; φ = 0.65).

**Figure 2:**
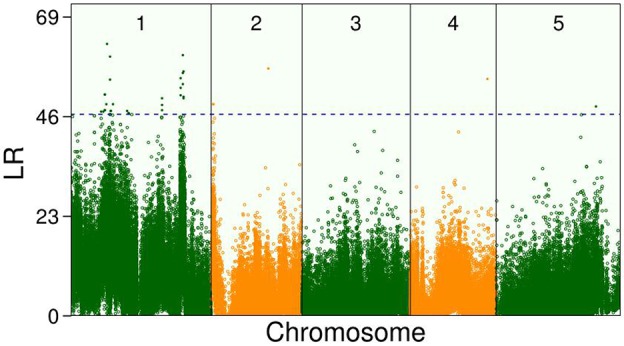
Manhattan plots of significance tests for single nucleotide polymorphisms (SNPs) across five *Arabidopsis* chromosomes by new model for growth curve of leaf number and dry weight of whole plant in the recombinant inbred lines (RILs). Dotted horizontal lines is the critical thresholds at the 5% significance level obtained after permutation test.

In the climate chamber, Ler exhibited a greater increase in leaf number than Sha but the dry weight showed a different trend. In addition, the growth curves of leaf number and biomass allocation varied markedly among the RILs, suggesting the existence of QTLs. By incorporating Equation (5) into Equation (1), the new model identified some QTLs that control the overall growth trajectory of leaf number at 1–9 weeks, the whole-plant dry weight, or both (Figure 2). The threshold value for the existence of QTLs obtained by 100 permutation tests was 47 at *P* = 0.05. Over two-thirds of the QTLs were within or adjacent to candidate genes with functional annotations (Table S1). Notably, most QTLs were located on chromosome1 (Figure 2) and some existed in the same candidate gene.

We selected the 10 most significant QTLs, all of which were annotated (Table 1). The SNPs chr1/24264668, chr1/24264657, and chr1/24264642 were located in a region of *CBSX6* related to vacuole function. The SNP chr1/24405170 was in *AS2* (related to leaf shape), which plays an important role in early development of leaf in *Arabidopsis*. The SNP chr1/23726087 was in the region of *AtMYB103*, which encodes an R2R3-type MYB transcription factor that regulates normal anther and pollen development in *Arabidopsis*. The SNPs chr1/7856114 and chr1/8513050 were annotated as hypothetical proteins, and their biological function is unknown.

**Table 1:** Physical positions, heritability, and function annotation of 10 significant single nucleotide polymorphisms (SNPs), detected by the new model that affect leaf number growth and the whole weight in *Arabidopsis*.

**SNP.ID**	**Gene.ID**	**Chr**	**Position**	**Allele**	**Heritability**		**Annotation**
					**Leaf number**	**Weight**	
1	AT1G22250	1	7856114	A/G	10.620	0.720	*Hypothetical protein*
2	AT1G65320	1	24264668	A/G	9.358	1.697	*CBSX6*
3	AT1G24060	1	8513050	C/T	1.029	0.867	*Hypothetical protein*
4	AT2G28680	2	12308953	C**/**T	0.267	2.027	*CRA1*
5	AT1G65620	1	24405170	C/G	0.205	0.243	*AS2*
6	AT1G65320	1	24264642	A/G	1.441	0.034	*CBSX6*
7	AT1G63910	1	23726087	G/T	0.378	0.002	*AtMYB103*
8	AT4G35060	4	16690778	A/T	1.978	1.151	*HIPP25*
9	AT1G23920	1	8451057	A/C	1.610	0.438	–
10	AT1G65320	1	24264657	A/C	0.527	0.085	*CBSX6*

**Figure 3:**
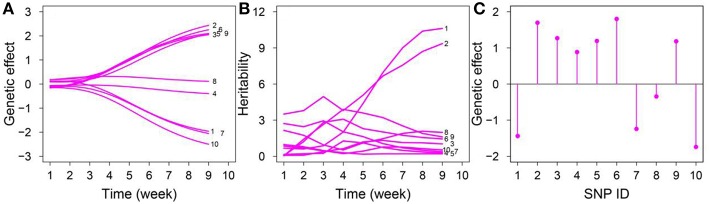
Temporal pattern of genetic effects for leaf number and dry weight and heritability for leaf number in an RIL of *Arabidopsis*. **(A)** Dynamic change of genetic effects of ten QTLs for leaf number. **(B)** Heritability explained by each of these chosen QTLs for leaf number. **(C)** Genetic effects of ten QTLs for the whole dry weight of *Arabidopsis*, detail information of SNP.ID is listed Table 1.

For each significant QTL, we estimated genotype-dependent growth and biomass accumulation parameters to assess the dynamic pattern of the genetic effects of growth phenotype and the distribution of genetic effects of static trait exerted by each locus (Figure 3). We also estimated the correlation coefficients for the growth of leaf number and dry weight. The magnitudes of the genetic effects on leaf number varied over time; five and three QTLs displayed ascending and decreasing trends, respectively (Figure 3A). The temporal variation in heritability explained by leaf number was observed for 10 SNPs (Figure 3B); chr1/7856114 and chr1/24264668 increased monotonously over time; chr2/12308953, chr1/24264642, chr1/23726087, and chr1/8451057 increased rapidly and subsequently decreased gradually, with a peak at weeks 2–4; chr1/24405170 decreased consistently over time. The magnitudes of the genetic effects of the 10 QTLs on dry weight are shown in Figure 3C.

### Test of Covariation QTLs

The advantage of our model lies in its capacity to test whether a significant QTL affects the genetic covariation between the dynamic change in a phenotype and biomass accumulation (Equations 7 and 8). We further tested whether the QTLs control two traits or only one of them using the 10 most significant QTLs. Most of the QTLs affected the dynamic change in leaf number but only two impacted dry weight; chr1/8513050 and chr1/8451057 affected both phenotypes. chr2/12308953 and chr4/16690778 did not influence either phenotype, but they controlled the covariation growth for two traits and so are *cov*QTLs. Therefore, the expression of a *cov*QTL is sensitive to the correlation between dynamic and static traits and may vary depending on the type of trait.

### Computer Simulation

We conducted a computer simulation to validate the statistical properties of our model based on the working example used in this study. Data were simulated by assuming that dynamic and static traits are either correlated or uncorrelated. The phenotype was determined by a set of QTLs among 1,000 simulated markers, plus a residual error following a multivariate normal distribution. In the correlated simulation scenario (Scenario 1, ϕ = 0.65; Scenario 2, ϕ = 0.4; Scenario 3, ϕ = 0.2), the phenotype of a growth trait over time was simulated by time-varying genotypic values (Equation 5) plus a covariance matrix (Equation 3), whereas phenotypic data in the uncorrelated simulation scenario (Scenario 4) were generated by neglecting φ. Scenarios 1, 2, and 3 indicate strong, moderate, and weak correlation, respectively, between the static and dynamic data. We adjusted the innovation variance to obtain curve heritability levels of 0.05 and 0.10. The simulation considers three sample sizes: 500, 200, and 100.

Data were analyzed reciprocally using analysis of variance (ANOVA), univariate functional mapping, and our new model. ANOVA was used to detect associations between molecular markers and static traits. Univariate functional mapping was used to identify specific QTLs that govern processes and patterns of development over time. As expected, traditional mapping (ANOVA and univariate functional mapping) detected significant QTLs from the uncorrelated data at a moderate sample size and heritability; however, its power for QTL detection among strongly and moderately correlated data was substantially lower (0.93–0.72; Table 2). Similarly, our new model showed reasonably good power to detect QTLs hidden within the correlated data, although its performance was dramatically slower for analyzing uncorrelated data at small and moderate sample sizes. The power of the new model was better than that of traditional approaches when correlation coefficient ϕ = 0.2 at large sample sizes. The performance of the new model was very similar to those of traditional methods for uncorrelated data at large sample sizes, with the same trends reflected in weakly correlated data at small and moderate sample sizes (Table 2). The results of the simulation studies suggest that the new model is suitable for related and unrelated cases at large sample sizes. The new model is also suitable for correlation coefficients ≥ 0.2 at small and moderate sample sizes.

**Table 2:** Power of QTL detection by traditional method (ANOVA and Functional mapping) and new model from the data simulated under four scenarios, respectively, under different heritabilities, 0.05 and 0.10, for sample size 500, 200, and 100.

		**Simulation**											
		**Scenario 1**						**Scenario 2**					
		***N*** **= 100**		***N*** **= 200**		***N*** **= 500**		***N*** **= 100**		***N*** **= 200**		***N*** **= 500**	
Method		*H*^2^ = 0.05	*H*^2^ = 0.1	*H*^2^ = 0.05	*H*^2^ = 0.1	*H*^2^ = 0.05	*H*^2^ = 0.1	*H*^2^ = 0.05	*H*^2^ = 0.1	*H*^2^ = 0.05	*H*^2^ = 0.1	*H*^2^ = 0.05	*H*^2^ = 0.1
	New model	60%	86%	76%	94%	92%	96%	58 %	81%	72%	91%	89%	95%
	Traditional	33%	63%	40%	72%	48%	77%	47 %	73%	60%	78%	62%	84%
		**Scenario 3**						**Scenario 4**					
		***N*** **= 100**		***N*** **= 200**		***N*** **= 500**		***N*** **= 100**		***N*** **= 200**		***N*** **= 500**	
Method		*H*^2^ = 0.05	*H*^2^ = 0.1	*H*^2^ = 0.05	*H*^2^ = 0.1	*H*^2^ = 0.05	*H*^2^ = 0.1	*H*^2^ = 0.05	*H*^2^ = 0.1	*H*^2^ = 0.05	*H*^2^ = 0.1	*H*^2^ = 0.05	*H*^2^ = 0.1
	New model	53%	80%	69%	86%	87%	95%	52 %	77%	65%	84%	84%	93%
	Traditional	53%	78%	71%	85%	76%	89%	59 %	84%	81%	93%	86%	95%

**Figure 4:**
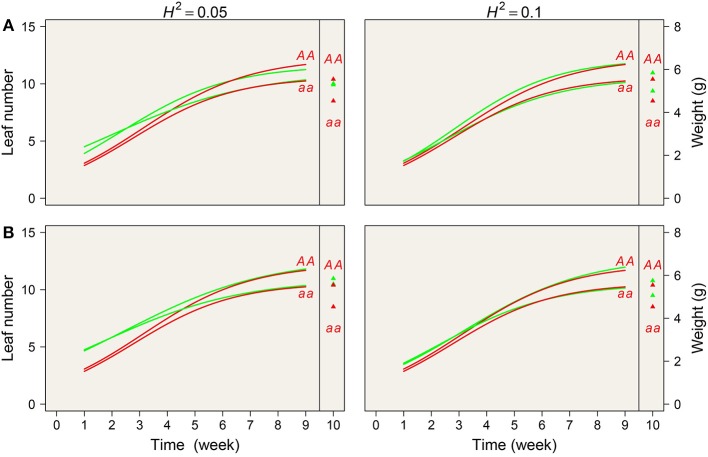
Simulation results by mimicking one significant QTL detected from the real example in terms of sample size (**A** represents *N* = 200 and **B** represents *N* = 100) and heritability (0.05 and 0.10). Estimated mean value and the true value are denoted by red and green lines, respectively.

We plotted estimated growth curves and static values from the simulated data; the true curves were within the confidence interval of the estimated curves (Figure 4). The above results suggest that our new model not only enables precise mapping of any QTL that affects genetic covariation in phenotypic traits but also that it is statistically robust for the identification of significant QTLs for a single phenotypic trait.

## Discussion

The growth and survival of any organism require maintenance of functional associations via the coordinated development of various organs. During the plant lifecycle, the covariant relationship between the growth and development of organs and biomass accumulation, such as leaf growth and dry weight, plays a pivotal role in photosynthesis and nutrition or water absorption and so on. Research to date has focused on the relationship between organ growth and biomass at the ecological scale, while the underlying genetic mechanisms have been neglected (Poorter and Nagel, 2000; Shipley and Meziane, 2002). GWASs enable elucidation of the genetic architecture of complex traits by identifying the genes that control phenotypic variation (Visscher et al., 2012). Because traits are dynamic, functional genome-wide association studies (*f* GWASs) use developmental processes to generate genotype-phenotype maps. Their use of mathematical equations for trait formation renders *f* GWASs statistically powerful and biologically relevant (Wang et al., 2018).

We performed an *f* GWAS to identify QTLs involved in plant organ growth and biomass allocation. Our model assesses not only the effects of genes on the variation in traits associated with organ or tissue growth and biomass accumulation but also the relationships of growth and biomass with pleiotropic genes or different genes in a strong linkage. Logistic equations are useful for biological research at the cell, organ, tissue, organism, and population levels. Combining logistic equations with the new model, we can better describe the forms of growth curve. By analyzing the GWAS data of recombinant inbred *Arabidopsis* lines, we identified 10 QTLs related to the change in leaf number or whole-plant biomass. The biological functions of some of these QTLs have been validated.

As shown in Equation (6), we assessed the genetic control of the QTLs. *CBSX6* (chr1/24264668, chr1/24264642, and chr1/24264657) encodes a cystathionine β-synthase family protein that affects thioredoxin activation, controls cellular H_2_O_2_ levels, and modulates plant development and growth (Ok et al., 2012). The association of *AS2* (chr1/23726087), which encodes a member of a family of proteins characterized by cysteine repeats and a leucine zipper, is involved in *knotted1-like homeobox* (*KNOX*) gene regulation (Hay and Tsiantis, 2010; Vial-Pradel et al., 2018). *KNOX* proteins are homeodomain transcription factors that maintain an important pluripotent cell population called the shoot apical meristem, which generates the entire aboveground body in vascular plants (Hay and Tsiantis, 2010). The transcription factor *AtMYB103* was previously identified as a member of the transcriptional network that regulates secondary wall biosynthesis in xylem tissues of *Arabidopsis*, and has been hypothesized to be involved in cellulose biosynthesis (Öhman et al., 2013).

Any QTL that controlled the two traits simultaneously was considered a *cov*QTL. We detected two *cov*QTLs by Equations (7) and (8), *CRA1* (chr2/12308953) and *HIPP25* (chr4/16690778), involved in plant structure, growth, and development. *CRA1* was expressed during flowering, petal differentiation, and expansion (Li et al., 2008; Sekhon et al., 2012), and in collective leaf structure, flower, petal, pollen, and sepal (Shapiguzov et al., 2016; Robinson et al., 2018). *HIPP25* was expressed in LP.04 at the four-leaves-visible stage and in LP.06 at the six-leaves-visible, flowering, petal differentiation, and petal expansion stages (Jiang et al., 2012; Ogawa et al., 2015; Xiao et al., 2018) and in the carpel, leaf apex, petal, plant embryo, pollen, root, sepal, stamen, stem, and leaf vasculature (Chauvin et al., 2013; Shapiguzov et al., 2016; Luo et al., 2018). These two *cov*QTLs are major contributors to leaf growth and biomass accumulation in *Arabidopsis*. Therefore, our new method enables elucidation of the genetic mechanisms that control growth and biomass accumulation in plants.

The results of our computer simulation studies show that our model has favorable statistical properties and is expected to produce reasonably accurate results, even when two traits are weakly correlated. At modest sample sizes, the power of the new model is lower than those of traditional methods due to rare variance between two datasets, mainly because covariance parameter estimates are not precise. However, the new method is expected to solve this issue at large sample sizes. The simulation results of our model will certainly improve experimental design aimed at exploring the genetic mechanisms involved in growth and biomass accumulation in plants.

Our method can be modified to increase its realism. Because it was based on a dynamic phenotype and a static trait, the ability of our method to make statistical inferences about the genetic basis of multiple growth traits and biomass allocation may be limited (Alimi et al., 2013; El-Soda et al., 2014). By simultaneously analyzing multiple traits, this issue can be resolved from framework of function mapping, such as proposing system mapping based on functional mapping (Wu et al., 2011). In addition, our new method was associated with single SNP, but the main-effects model is likely too simple to be used for characterizing genetic variants in quantitative traits (Pang et al., 2012; Mackay, 2014). We plan to integrate high-order QTL–QTL interactions into our new method. A multiple-QTL epistatic model will enhance our understanding of the contribution of epistatic effects to organ growth and biomass allocation. Since nearby loci can share similar covariance for the same set of traits, it is more efficient to perform hypothesis testing using a joint estimation of nearby loci. In future research, we will attempt to extend the new model to analyze, simultaneously, nearby markers.

## Author Contributions

JG derived the model, performed data analysis and simulation studies, and wrote R-based software. JG and YC participated in data collection. RW and LJ conceived the conceptual idea, supervised the study. JG and LJ wrote the manuscript.

### Conflict of Interest Statement

The authors declare that the research was conducted in the absence of any commercial or financial relationships that could be construed as a potential conflict of interest.

## Supplementary Material

Figure S1 The goodness of fit of growth equation to leaf number for each RIL.

Table S1 Gene annotations of significant SNPs detected by the new model.
