# Supplementary figures and images for: Mapping Covariation Quantitative Trait Loci That Control Organ Growth and Whole-Plant Biomass
Source: Front Plant Sci. 2019 Jun 4;10:719. doi: 10.3389/fpls.2019.00719 (PMC6558071; doi:10.3389/fpls.2019.00719)

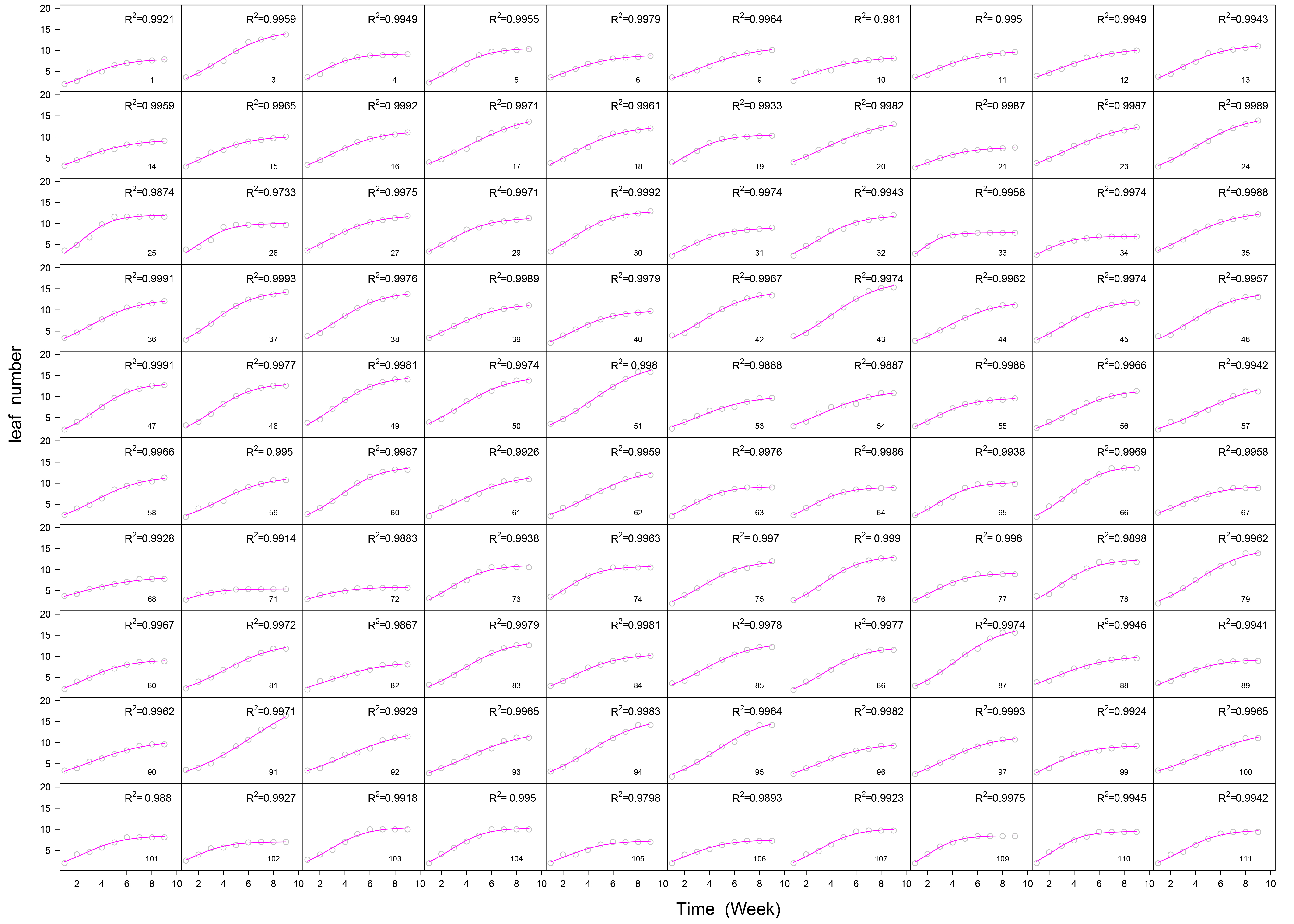

Supplement: Figure S1 — The goodness of fit of growth equation to leaf number for each RIL. [file Image_1.JPEG]
